# Gallbladder mucinous cystic neoplasm: Diagnostic challenges and multimodality imaging correlation

**DOI:** 10.1016/j.radcr.2026.02.046

**Published:** 2026-03-14

**Authors:** Rabita Alamgir, Nikhil Kanthala, Arvind Sabesan, Randi LaPoint, Mandip Gakhal

**Affiliations:** a Department of Radiology, ChristianaCare, 4755 Ogletown Stanton Road, Newark, DE 19718, USA; b Surgical Oncology, Helen F. Graham Cancer Center, 4701 Ogletown Stanton Road, Newark, DE 19713, USA; c Department of Pathology, ChristianaCare, 4755 Ogletown Stanton Road, Suite L130, Newark, DE 19718, USA

**Keywords:** Mucinous cystic neoplasm, Gallbladder, Ultrasonography, MRCP, ERCP, Mirizzi syndrome

## Abstract

Mucinous cystic neoplasm of the gallbladder is a rare, though generally benign, entity typically diagnosed following cholecystectomy due to its nonspecific clinical presentation and imaging characteristics. This case report describes an unusual presentation of mucinous cystic neoplasm of the gallbladder mimicking a multiseptated gallbladder, wherein a 41-year-old female presented with biliary obstruction. Imaging studies revealed a gallbladder with features resembling a multiseptated gallbladder, a rare congenital variant. Consequently, the patient was scheduled for elective cholecystectomy. Intraoperative findings and histopathological examination of the resected gallbladder unexpectedly revealed a large mucinous tumor of the gallbladder and common bile duct. This case underscores the importance of considering neoplastic etiology in the differential diagnosis of atypical gallbladder imaging findings, especially in the presence of biliary obstruction, and highlights the critical role of histopathology in establishing a definitive diagnosis. Early identification and appropriate surgical intervention are essential for improving patient outcomes.

## Introduction

Mucinous cystic neoplasms (MCNs) of the gallbladder are extremely rare tumors hypothesized to represent 0.02% of all hepatobiliary MCNs [1]. Hepatobiliary MCNs are diagnosed at a mean age of 45 years if noninvasive and 59 years if there is an invasive component [2]. They are reported more frequently in women than men, with most patients presenting with right upper quadrant pain [3]. The infrequency of these tumors and the nonspecific nature of their clinical presentations make it challenging to distinguish them from other gallbladder pathologies or anomalies.

With approximately 90% characterized as benign, MCNs often reach greater sizes before manifesting with symptoms of biliary obstruction [3,4]. MCNs also exhibit a unique histological structure characterized by an inner mucinous epithelial layer and an outer ovarian-type stromal layer [2,[4], [5]]. While imaging techniques such as computed tomography (CT) and magnetic resonance imaging (MRI) may suggest its inclusion in the differential diagnosis, definitive diagnosis relies on histopathology.

In this report, we present a case of a patient with Mirizzi-like syndrome secondary to MCN of the gallbladder. Histopathology following cholecystectomy confirmed MCN of the gallbladder communicating with the cystic, common hepatic, and common bile ducts.

## Case report

A 41-year-old woman presented to the hospital with a 1-week history of painless jaundice. Past medical history included rheumatoid arthritis and systemic lupus erythematosus. She had no prior surgical history or relevant family history. Physical examination revealed jaundice without other remarkable findings. Laboratory data demonstrated an elevated aspartate aminotransferase of 122 IU/L (reference range, 0-40 IU/L), alanine aminotransferase of 212 IU/L (reference range, 0-32 IU/L), alkaline phosphatase of 336 IU/L (reference range, 44-121 IU/L), gamma-glutamyl transpeptidase of 163 IU/L (reference range, 0-60 IU/L), total bilirubin of 6.6 mg/dL (reference range, 0-1.2 mg/dL), direct bilirubin of 4.99 mg/dL (0-0.4 mg/dL), and indirect bilirubin of 1.61 mg/dL (reference range, 0.1-0.8 mg/dL). Lipase and amylase levels were within normal limits, arguing against pancreatitis. Antimitochondrial (AMA-M2) antibody was obtained and was negative (<20 U) (Table 1). Overall, these results supported a mechanical rather than inflammatory etiology for the patient’s cholestatic laboratory profile.

**Table 1:** Laboratory values at presentation.

Parameter	Result	Reference range
CBC		
WBC (×10³/µL)	5.9	3.4-10.8
Hgb (g/dL)	13.1	11.1-15.9
Hct (%)	37.4	34.0-46.6
Plt (×10³/µL)	296	150-450
Coagulation		
INR	0.9	0.9-1.2
Liver function tests		
Alb (g/dL)	4.2	3.9-4.9
AST (U/L)	122	0-40
ALT (U/L)	212	0-32
ALP (U/L)	336	44-121
GGT (U/L)	163	0-60
T. bili (mg/dL)	6.6	0-1.2
D. bili (mg/dL)	4.99	0-0.4
Pancreatic enzymes		
Lipase (U/L)	13	13-60
Amylase (U/L)	58	30-110
Autoimmune profile		
AMA-M2 (U)	<20.0	a
Viral serologies		
Hep A IgM	Negative	–
Hep B core Ab IgM	Negative	–
Hep B surface Ag	Negative	–
Hep C Ab	Nonreactive	–

Alb, albumin; ALP, alkaline phosphatase; ALT, alanine aminotransferase; AMA-M2, anti-mitochondrial antibody M2 subtype.AST, aspartate aminotransferase; D. bili, direct bilirubin; GGT, gamma-glutamyl transferase; Hct, hematocrit; Hep, hepatitis; Hgb, hemoglobin; INR, international normalized ratio; Plt, platelet count; T. bili, total bilirubin; WBC, white blood cell count.

a Values <20 U for AMA-M2 are considered negative.

### Ultrasound findings

**Fig. 1:**
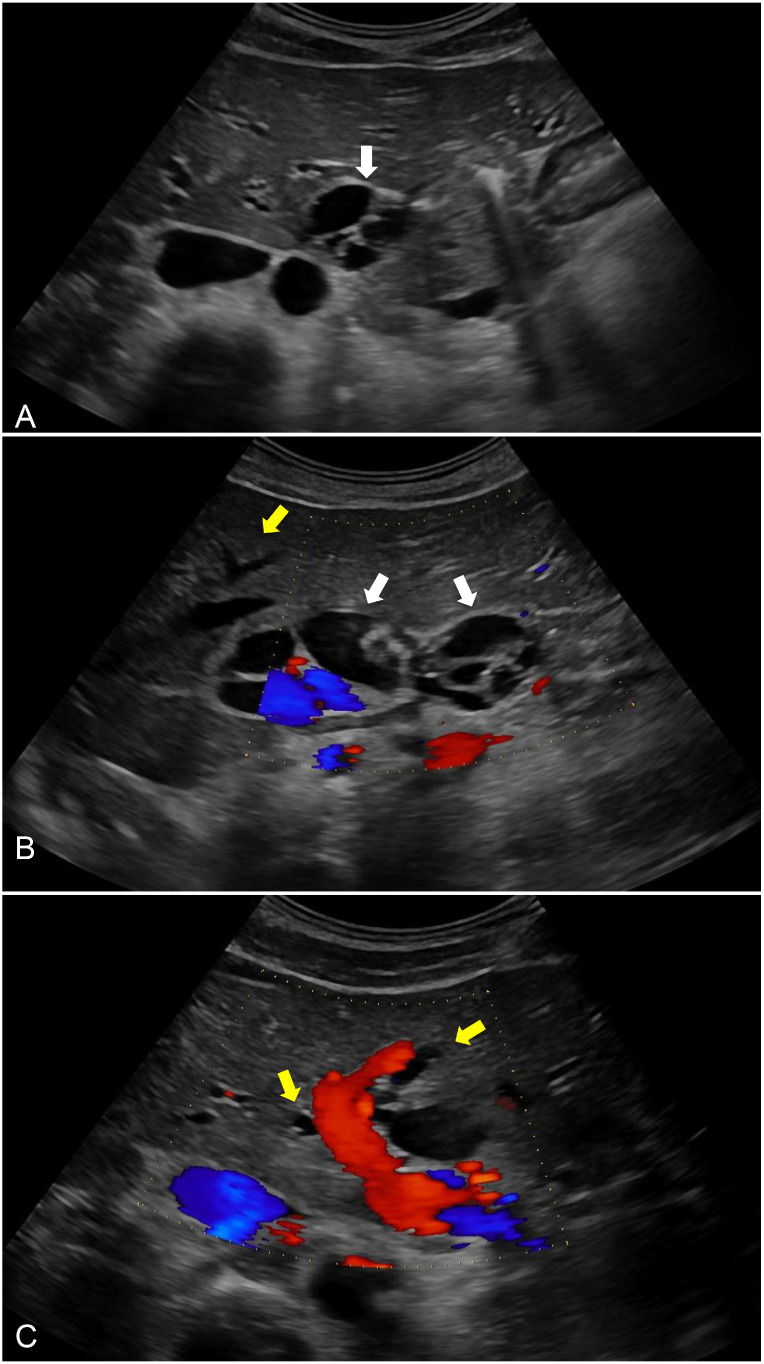
Ultrasound of the abdomen: (A) Transverse decubitus grayscale ultrasound of the gallbladder demonstrates a multiseptated cystic lesion (white arrow) within the gallbladder fossa. (B) Sagittal decubitus ultrasound of the gallbladder with color Doppler shows no internal vascularity within the lesion (white arrows) and associated intrahepatic bile duct dilation (yellow arrow). (C) Color Doppler evaluation of the main portal vein demonstrates preserved flow. There is intrahepatic and extrahepatic bile duct dilation (yellow arrows).

Abdominal ultrasonography demonstrated a multiloculated cystic structure within the gallbladder fossa containing internal septations, possibly with few hyperechoic foci (Figs. 1A and B). There was also dilation of the intrahepatic and extrahepatic bile ducts, for which further evaluation with MRI with magnetic resonance cholangiopancreatography (MRCP) was recommended (Fig. 1C).

### MRI and magnetic resonance cholangiopancreatography findings

**Fig. 2:**
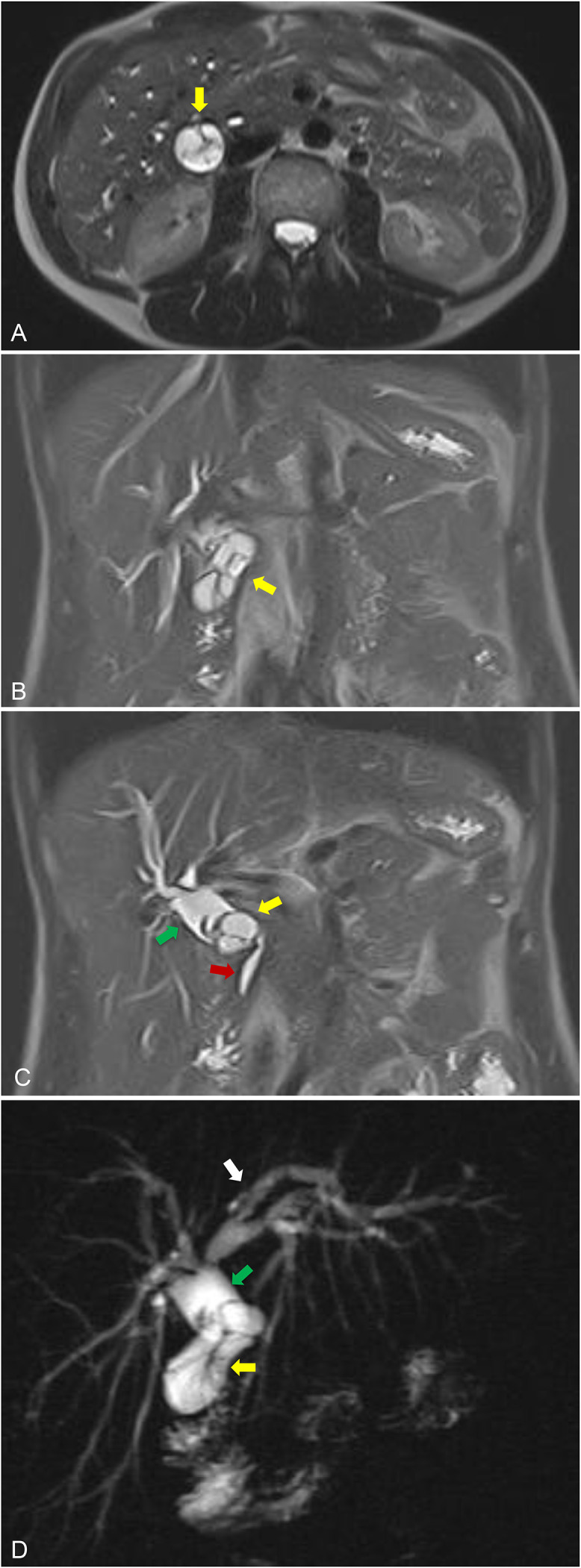
MRI/magnetic resonance cholangiopancreatography (MRCP) of the abdomen: (A) Transverse half Fourier single shot turbo spine echo MR image demonstrates a cystic lesion arising from the gallbladder with internal septations (yellow arrow). (B and C) Coronal half Fourier single shot turbo spine echo MR images demonstrate multiseptated gallbladder lesion (yellow arrows) and associated intrahepatic biliary ductal dilation (white arrow) and common hepatic ductal dilatation (green arrow). There is no significant common bile duct dilatation (red arrow). (D) Heavily T2 weighted thick slab coronal MRCP image demonstrates a multiseptated gallbladder lesion (yellow arrow) as well as intrahepatic (white arrow) biliary ductal and common hepatic (green arrow) ductal dilation.

MRI with MRCP revealed a multiseptated cystic structure in the expected region of the gallbladder measuring up to approximately 4.4 × 2.5 × 3.8 cm^3^ with associated intrahepatic and partial extrahepatic ductal dilatation (Figs. 2A–D). The common hepatic duct measured up to 18 mm, indicating significant biliary ductal dilatation. Based on imaging and laboratory data, the patient was then referred for an endoscopic retrograde cholangiopancreatography (ERCP).

### Endoscopic retrograde cholangiopancreatography findings

**Fig. 3:**
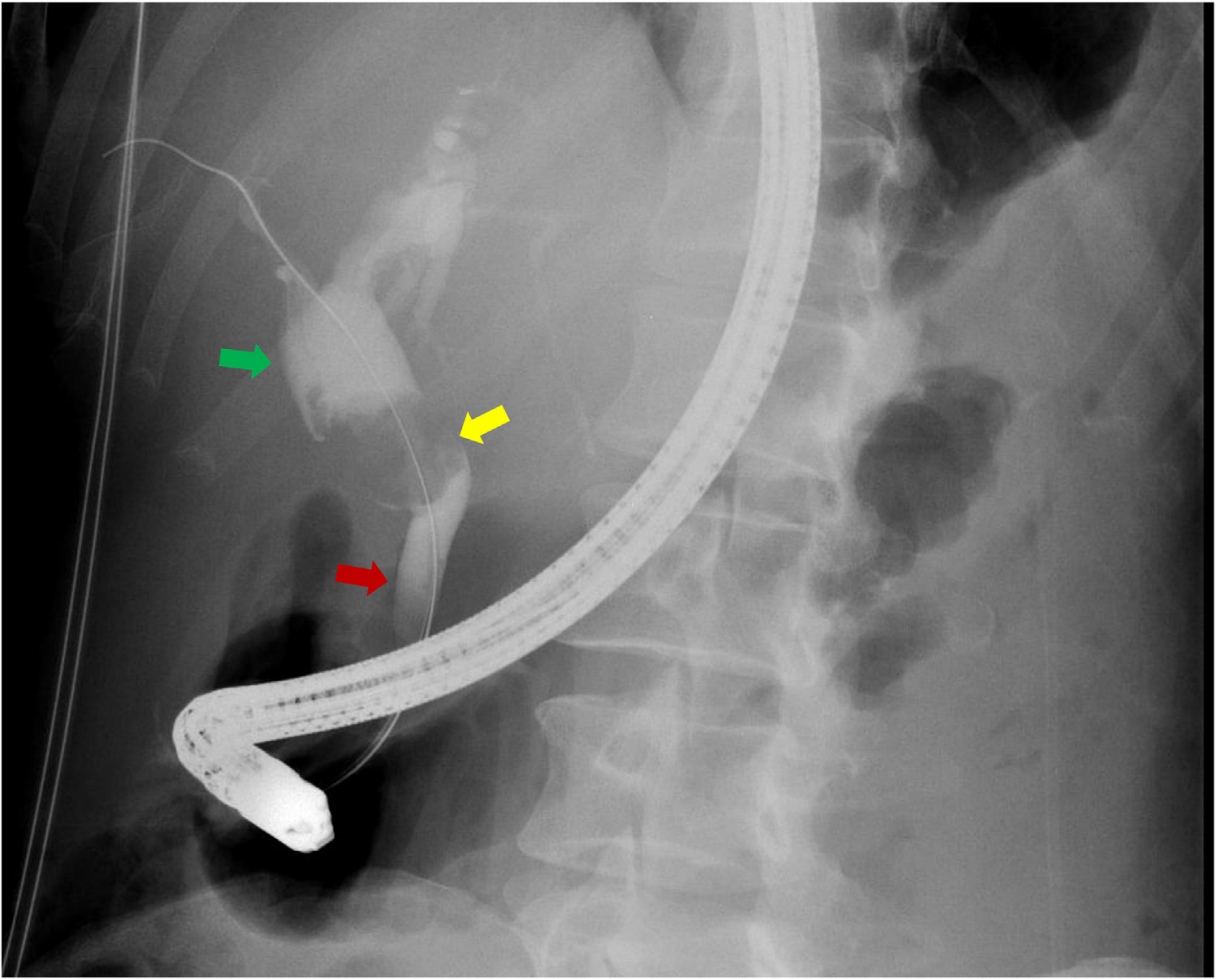
Endoscopic retrograde cholangiopancreatography (ERCP): Radiographic image from ERCP procedure demonstrates superior common hepatic duct dilatation (green arrow) and mass-like filling defect and narrowing produced by the gallbladder at the level of the expected confluence of the common hepatic duct, cystic duct, and common bile duct (yellow arrow), with the common bile duct opacified further inferiorly (red arrow).

ERCP demonstrated mass-like extrinsic compression and filling defect reflecting the gallbladder into the common bile duct and common hepatic duct, with upstream dilation of the common hepatic duct, raising concern for Mirizzi syndrome (Fig. 3). An associated stenosis of the mid common bile duct was also identified. A biliary sphincterotomy was subsequently performed, and a biliary stent was placed into the left hepatic duct.

### CT findings

**Fig. 4:**
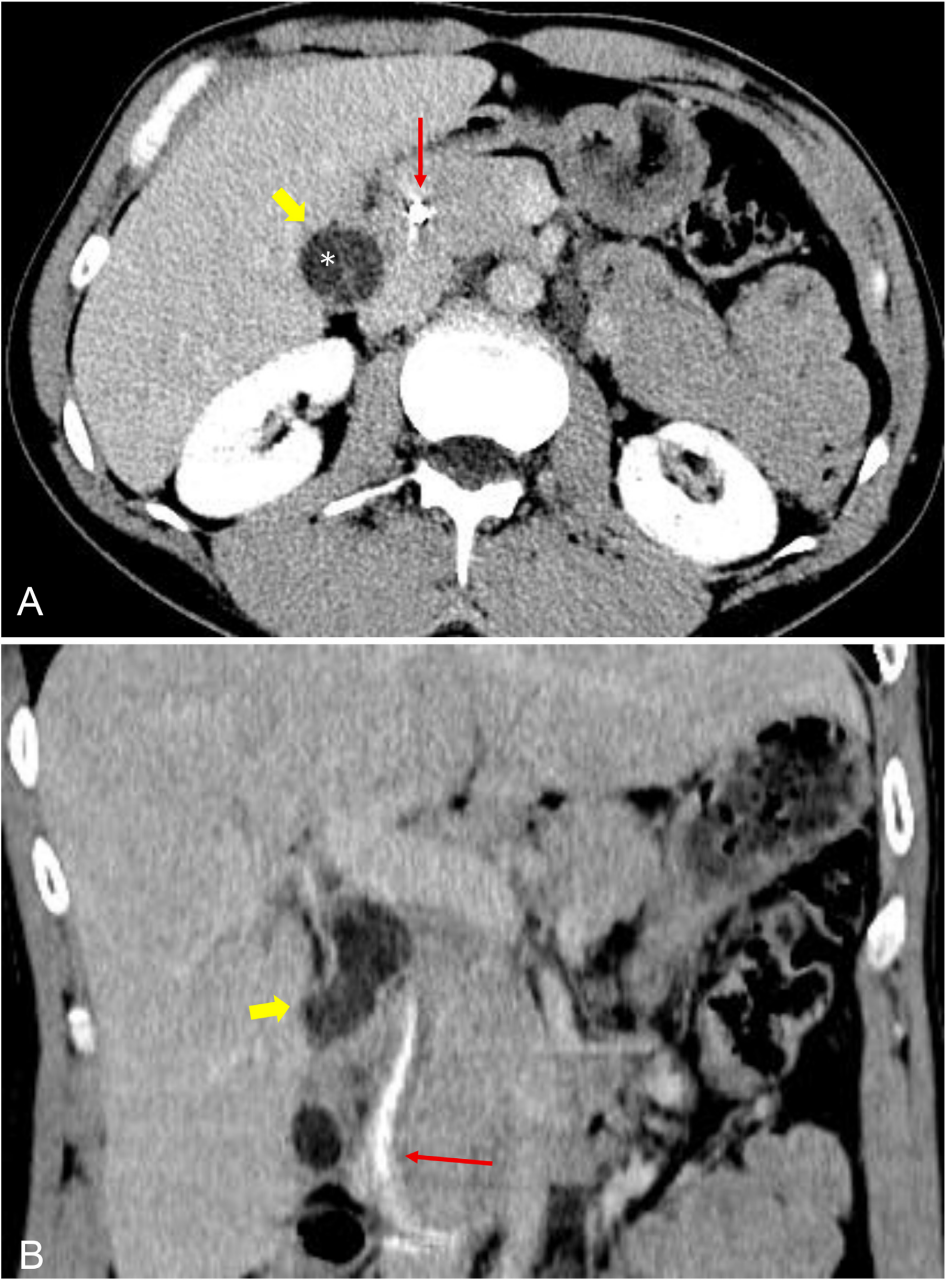
CT of the abdomen, contrast enhanced, portal venous phase: (A) Transverse CT image demonstrates the gallbladder (yellow arrow) with internal septations (asterisk) and adjacent biliary stent (red arrow). No mural nodularity, soft tissue attenuation mass, or internal calcification is demonstrated. (B) Coronal CT image demonstrates a multiseptated lesion within the gallbladder (yellow arrow) and a biliary stent (red arrow).

CT of the abdomen was then obtained due to the patient’s worsening abdominal pain 1 week after the ERCP. CT showed a biliary stent in situ and a cystic lesion in the gallbladder region with septations (Figs. 4A and B).

### Operative and histopathological findings

**Fig. 5:**
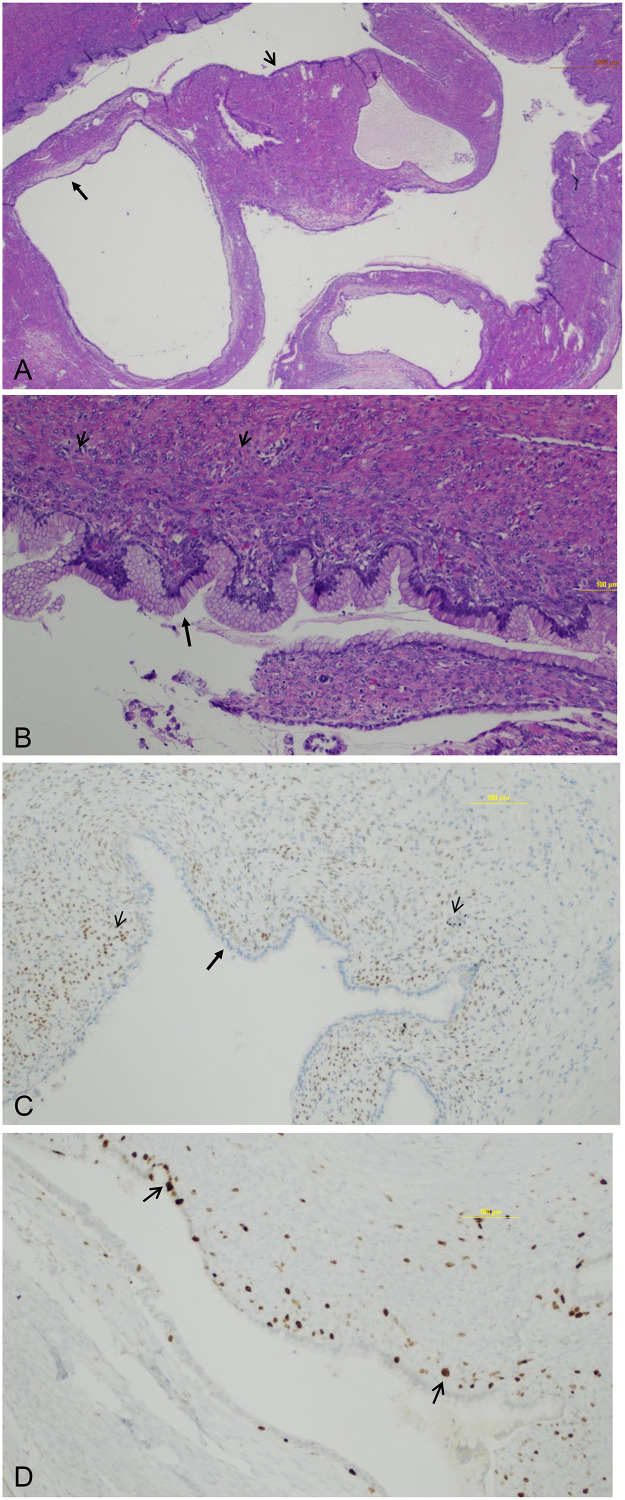
Histopathology. (A) Hematoxylin and eosin (H&E) stained section at low magnification (2×) demonstrates a multiloculated cystic lesion lined by mucinous epithelium (arrows) and supported by a densely cellular subepithelial stromal layer (arrowheads). (B) H&E stain at higher magnification (10×) shows tall columnar mucin-secreting epithelial cells (arrows) overlying ovarian-type stroma composed of spindle-shaped cells (arrowheads). The epithelial nuclei are uniformly sized and basally located, without nuclear stratification or mitotic figures, confirming the absence of cytologic atypia or invasive features. (C) Estrogen receptor (ER) immunohistochemical stain (10×) demonstrates strong nuclear positivity within the stromal component (arrowheads), confirming ovarian-type stroma. In contrast, the epithelial lining at the tissue periphery remains pale and unstained (arrow), indicating absence of ER expression. (D) Ki-67 immunostain (10×) shows a low proliferative index, with only rare positively staining nuclei (arrowheads), consistent with benign behavior.

Open cholecystectomy with resection of the common hepatic and bile ducts was performed. Intraoperatively, a neoplasm extended from the gallbladder infundibulum to the mid portion of the common bile duct. The specimen sent to pathology consisted of an 11.5 × 3.7 × 2.7 cm^3^ gallbladder with a pink-red and edematous outer surface. The gallbladder was filled with colorless mucinous fluid as well as a 3.5 × 2.0 × 1.5 cm^3^ pedunculated lesion occluding the cystic duct and extending into the common bile duct. There was no evidence of cholelithiasis. The lesion was then sectioned to reveal a multilocular cystic structure without solid components. Histopathological diagnosis confirmed the presence of MCN involving the gallbladder and cystic duct without evidence of dysplasia or carcinoma (Figs. 5A–D). The patient had an uneventful postoperative course.

## Discussion

MCN of the gallbladder is an exceptionally rare entity that has only been described in a few published case reports. The earliest documented case dates to 1901, when Bishop reported a 40-year-old female with a cystic lesion of the gallbladder [6]. Microscopically, MCN of the gallbladder is characterized by multiloculated cysts lined by columnar, cuboidal, or flattened biliary-type or mucinous epithelial cells. A defining histopathologic feature is the presence of ovarian-type stroma beneath the epithelial lining, composed of densely packed spindle-shaped or oval cells. This stromal component is crucial for distinguishing MCN from other cystic lesions [7,8].

In a study by Devaney et al., among 52 cases of hepatobiliary MCN, 85% occurred in the liver, and only one case (0.02%) arose in the gallbladder. Furthermore, 96% of MCNs without associated dysplasia occurred in women [7]. The clinical manifestations of nondysplastic MCNs vary according to tumor size and location, but most patients present with right upper quadrant pain [8,9].

When evaluating suspected hepatobiliary disease, laboratory findings can provide important diagnostic clues. Abnormal liver function tests, especially those demonstrating cholestasis, are frequently reported in MCN [3]. Ultrasonography is the preferred initial imaging modality in this setting. On sonography MCN typically appears as a unilocular or multilocular cystic mass with internal septations. Echogenic debris may reflect mucin, blood products, or proteinaceous material. Posterior acoustic shadowing, suggestive of septations or calcifications, may also be present, though this was not seen in our case [1].

Further cross-sectional imaging can help delineate the lesion’s morphology and its relationship to surrounding structures. CT may demonstrate mural or septal calcifications or enhancing soft tissue components, neither of which were present in our patient. MRI with MRCP typically confirms a multiseptated cystic mass with variable signal intensity depending on the fluid content, as well as associated intrahepatic or extrahepatic ductal dilatation. In our case, MRCP revealed ductal dilatation with Mirizzi syndrome type appearance. Endoscopic ultrasound and ERCP can further clarify communication between the lesion and the biliary system. Our patient’s endoscopic ultrasound demonstrated gallbladder impingement on the common hepatic and common bile ducts, also suggesting Mirizzi syndrome. Given the multiseptated cystic appearance on preoperative imaging, a multiseptated gallbladder (MSG) was initially favored as the diagnosis.

The differential diagnosis for cystic gallbladder masses includes hydatid cyst, abscess, adenomyomatosis, mucinous cystadenocarcinoma, and MCN. Hydatid cysts and abscesses are generally excluded based on clinical presentation and laboratory results. Adenomyomatosis can be excluded by the absence of echogenic foci or reverberation artifacts on ultrasound [10]. Gallbladder carcinoma is often associated with gallstones, porcelain gallbladder, or an enhancing soft tissue mass, none of which were identified in the presented patient [11]. When a MCN demonstrates invasive carcinoma, the lesion is termed cystadenocarcinoma. Although differentiating mucinous cystadenoma from cystadenocarcinoma is often challenging, markedly elevated carbohydrate antigen 19-9 (CA 19-9) levels (absent in our case) have been reported as a helpful indicator of malignancy [12].

MSG, also referred to as septate or honeycomb gallbladder, is also a consideration in the differential diagnosis, with approximately 150 cases reported worldwide [13]. First recognized radiologically by Knetsch in 1952 and later characterized pathologically by Simon and Tandon [14], MSG is a congenital malformation in which thin septae divide the gallbladder lumen into multiple communicating chambers. It is thought to result from incomplete vacuolization of the gallbladder bud during development [15]. Acquired or pseudomultiseptated gallbladder, though rare, may occur secondary to chronic inflammation, cholelithiasis, or organized hemorrhage [13,16]. Sasaki et al. described two such acquired cases in which the septations lacked smooth muscle fibers, helping distinguish them from congenital forms [13,16]. Similar to MCN, MSG often presents with nonspecific or incidental findings on imaging [15]. The overlap in imaging features between MCN and MSG led to the preoperative presumption of an MSG.

In the presented case, persistent cholestatic laboratory values and recurrent symptoms despite biliary stent placement warranted definitive surgical intervention. Fine-needle aspiration for cytologic analysis yielded limited diagnostic information, consistent with the known low cellularity of cystic lesions. Following cholecystectomy and biliary reconstruction, histopathological evaluation established the diagnosis of MCN of the gallbladder extending into the biliary tree. Surgical resection provided lasting symptomatic relief.

In summary, this case highlights that MCNs of the gallbladder can closely mimic MSG on imaging. Moreover, MCNs of the gallbladder and biliary tree may present with biliary obstruction or even Mirizzi-like syndrome, underscoring the importance of maintaining a broad differential and confirming the diagnosis histopathologically.

## Conclusion

Gallbladder MCN is a rare but important diagnostic consideration when evaluating cystic gallbladder lesions, including those with biliary obstruction. This case highlights a potential diagnostic imaging pitfall wherein a gallbladder MCN mimicked an MSG. Use of multimodality imaging and radiologic–pathologic correlation are essential to ensure accurate diagnosis, along with timely surgical management.

## Patient consent

Written informed consent was obtained from the patient for publication of this case report and accompanying images.
